# In Vitro and In Vivo Evaluation of PEGylated Starch-Coated Iron Oxide Nanoparticles for Enhanced Photothermal Cancer Therapy

**DOI:** 10.3390/pharmaceutics13060871

**Published:** 2021-06-12

**Authors:** Reeju Amatya, Seungmi Hwang, Taehoon Park, Kyoung Ah Min, Meong Cheol Shin

**Affiliations:** 1 College of Pharmacy and Research Institute of Pharmaceutical Sciences, Gyeongsang National University, 501 Jinju Daero, Jinju 52828, Gyeongnam, Korea; reejuamatya94@gmail.com (R.A.); xornf@naver.com (T.P.); 2 College of Pharmacy and Inje Institute of Pharmaceutical Sciences and Research, Inje University, 197 Injero, Gimhae 50834, Gyeongnam, Korea; hsm8549@naver.com

**Keywords:** iron oxide nanoparticle, polyethylene glycol, plasma half-life, photothermal therapy, cancer

## Abstract

Iron oxide nanoparticles (IONPs) possess versatile utility in cancer theranostics, thus, they have drawn enormous interest in the cancer research field. Herein, we prepared polyethylene glycol (PEG)-conjugated and starch-coated IONPs (“PEG–starch–IONPs”), and assessed their applicability for photothermal treatment (PTT) of cancer. The prepared PEG–starch–IONPs were investigated for their physical properties by transmission electron microscopy (TEM), energy dispersive spectroscopy (EDS), X-ray diffraction (XRD), Fourier transform infrared (FT-IR) spectroscopy, and dynamic light scattering (DLS). The pharmacokinetic study results showed a significant extension in the plasma half-life by PEGylation, which led to a markedly increased (5.7-fold) tumor accumulation. When PEG–starch–IONPs were evaluated for their photothermal activity, notably, they displayed marked and reproducible heating effects selectively on the tumor site with laser irradiation. Lastly, efficacy studies demonstrated that PEG–starch–IONPs-based PTT may be a promising mode of cancer therapy.

## 1. Introduction

Photothermal therapy (PTT) is a local treatment modality that relies on the activation of light-absorbing agents by electromagnetic radiation, such as pulsed laser irradiation at the near-infrared (NIR) wavelength [1]. Upon laser irradiation, those photo-responsive materials could convert light to heat with high efficiency [2,3]. Thus, by focal application of laser to a specific diseased site after pre-delivery of the photoactive materials, thermal treatment could be available [4]. With the help of the recent discovery of many photoactive nanomaterials (including the IONPs), the PTT has become a hot issue, specifically, in the nanoparticle-based cancer research field [5,6,7,8].

Iron oxide nanoparticles (IONPs), clinically available MRI contrast agents, have drawn great interest in the pharmaceutical research fields [9,10]. For example, there are the following clinically used IONPs for MRI of the liver: ferumoxides (Feridex^®^ in USA), with a size of 120 to 180 nm, coated with dextran, and ferucarbotran (Resovist^®^), with a size of about 60 nm, coated with carboxydextran [11]. As important physical factors, the size and surface chemistry of IONPs have been known to play an important role in the interaction between particles and cellular structures. For using IONPs in clinical applications, it is important to evaluate the particle sizes and surface properties in determining the in vivo biocompatibility and toxic potential of magnetic IONPs. Nanoparticles have a high surface area-to-volume ratio, allowing more interaction among particles, or between particles and cells. It has been reported that the hydrodynamic sizes of particles should be small (<200 nm) to avoid uptakes by RES (liver or spleen filtration) in vivo, whereas the size should be >10 nm to escape from kidney filtration for prolonged blood circulation [12]. Moreover, Panyam et al. studied the effects of the hydrodynamic size distribution of nanoparticles on cytotoxicity, in results, suggesting that the cytotoxic effects evolved into a necrotic mechanism with nanoparticles at around 400 nm [13].

Proper application of surface-coating materials can stabilize IONPs, avoid agglomeration, and reduce toxicity issues [14]. The coating materials for the surface modification of IONPs need to be biocompatible and biodegradable to avoid nonspecific adsorption of proteins in blood and unnecessary immune responses after the systemic administration. To develop cancer therapeutic reagents based on IONPs, it is suggested to apply an appropriate coating to stabilize the iron oxide cores in an aqueous environment by electrostatic repulsion or steric hindrance [15]. In many studies, hydrophilic polymers, such as polyethylene glycol (PEG) or polyvinyl alcohol (PVA), have been used for the surface modification of IONPs, stably forming particle dispersion in blood and increasing half-life in vivo [16]. Surface charge also influences plasma protein binding, which in turn affects the in vivo biodistribution and clearance of IONPs from the circulation [17]. Biodegradable polymers, such as dextran and carbohydrate derivatives, are traditionally used as coating biomaterials for clinical preparations of IONPs [6,14]. These IONPs, with neutrally charged carbohydrates (dextran or starch), are relatively less toxic and have been well-tolerated in clinical use [16,18].

Apart from the low toxicity, the IONPs possess a unique property, which is magnetic susceptibility [10,19]. By focal application of an external magnet, the administered IONPs could be remotely directed to a target organ while circulating in the bloodstream [10,20]. Therefore, they have been considered to be not only a diagnostic agent, but also a highly favored magnetic-based theranostic agent [21,22]. Recently, other notable characteristics of the IONPs have gained many researchers’ attention, which is their high photo-thermal conversion capacity [7]. The IONPs could absorb light in the region of NIR wavelength, and transform it to heat. Because of this ability, the IONPs have also emerged as a potent PTT agent [23,24]. In contrast to other metallic nanoparticles (silver, gold, or copper), carbon nanotubes, or graphenes that hold photothermal properties, but are not readily biodegradable, the IONPs have been recognized as a safer PTT agent [25]. This is because the IONPs have excellent biodegradability in the in vivo environment and the iron ions released from the degraded IONPs could be assimilated by the body via a tightly regulated physiological homeostasis [26,27].

For effective, but safe, cancer PTT with the IONPs, there remain critical challenges to overcome. One of the challenges would be how to irradiate laser selectively to the tumor tissue, while the other is to accomplish high, but selective, tumor accumulation of the IONPs [28]. Specifically, to address the latter delivery issue, it would be necessary to develop long-circulating and stable IONPs that possess high photoactive properties [29]. PEG is a hydrophilic, biocompatible, and non-antigenic polymer used to stabilize magnetic particles [30]. There have been several studies to prepare functionalized magnetic nanoparticles by PEG, and those particles have been tested for cancer therapy. For example, various cancer treatment studies have been performed using magnetic nanoparticles modified by using a silane functionalized PEG [31], or adding sodium oleate and PEG as surfactants [32]. Due to the advantages of using PEG for functionalizing nanoparticles, it has been important to develop a process of modifying the surface of magnetic nanoparticles with different types of PEG, and testing those particles for cancer treatment. Cole et al. applied PEGylation to the starch-crosslinked IONPs, and they proved that better in vivo pharmacokinetic and magnetic targeting profiles for tumors could be achieved by the PEGylation [29]. However, the therapeutic benefits of the PEGylation on PTT have not been fully investigated yet.

In this research, we applied the stable chemical conjugation procedure to prepare polyethylene glycol (PEG)-conjugated and starch-coated IONPs (PEG–starch–IONPs), and fully characterized the physicochemical and photothermal properties. Furthermore, their applicability for PTT was evaluated in vitro and in vivo. The study results suggested that PEG–starch–IONPs could serve as an effective PTT agent for the treatment of cancer.

## 2. Materials and Methods

### 2.1. Materials

Starch-coated IONPs (“Starch-IONPs”) was purchased from Chemicell GmbH (Berlin, Germany). Ammonium hydroxide and epichlorohydrin were purchased from Sigma-Aldrich (St. Louis, MO, USA). Methoxy PEG Succinimidyl Carboxymethyl Ester (PEG-SCM; 5 kDa) was obtained from JenKem Technology USA (Plano, TX, USA).

### 2.2. Preparation of PEGylated Starch-Coated Iron Oxide Nanoparticles (PEG–Starch–IONPs)

The preparation of PEG–starch–IONPs was performed by the procedures of Zhang et al. [33]. Briefly, starch–IONP suspension (1 mL of 25 mg Fe/mL) was incubated with 1.3 mL of NaOH (6 M) for 10 min and then further incubated overnight (o.n.) after the addition of 0.7 mL of epichlorohydrin (crosslinker) at room temperature (RT). The crosslinked starch–IONPs were extensively dialyzed against double distilled water (DDW) and then aminiated by the addition of 1 mL of 30% ammonium hydroxide and incubation o.n. at RT. The aminated starch–IONPs were also extensively dialyzed against DDW. After then, the aminated starch–IONPs were PEGylated by incubation with PEG-SCM (5 kDa) 50 mg for 4 h at RT. The unconjugated PEG-SCM were removed by dialysis (molecular weight cut-off: 100 kDa). The final PEG–starch–IONP suspension was concentrated by using a Dynal magnetic separator (Invitrogen, Carlsbad, CA, USA) to 10 mg Fe/mL and kept in the refrigerator until further use.

### 2.3. Physical Characterization of PEG–Starch–IONPs

The iron (Fe) contents of PEG–starch–IONPs were analyzed by inductively coupled plasma optical emission spectroscopy (ICP-OES) using an Optima 5300DV spectrometer (Perkin Elmer, Norwalk, CT, USA). The amine and PEG contents were quantified using ninhydrin assay [34] and barium iodide assay [35], respectively. The morphology and size of PEG–starch–IONPs were examined by a transmission electron microscopy (TEM) (Tecnai 12, FEI Co., Hillsboro, OR, USA). The morphology and elements of PEG–starch–IONPs were investigated using a high-resolution TEM (HR-TEM) with energy dispersive spectroscopy (EDS) (Tecnai TF30 ST, FEI Co., Hillsboro, OR, USA). Crystallinity of cores in nanoparticles were confirmed by X-ray diffraction (XRD) (Bruker APEX2 diffractometer, Bruker, Billerica, MA, USA). The zeta potential and hydrodynamic size of PEG–starch–IONPs were measured using a dynamic light scattering instrument (DLS) (Zetasizer Nano ZS, Malvern Panalytical Ltd., Malvern, UK). To verify the PEGylation, FT-IR spectrophotometry was conducted (VERTEX 80v, Bruker, Billerica, MA, USA). The plasma stability of PEG–starch–IONPs was assessed by incubation of PEG–starch–IONPs in 10% plasma and the size difference along the time was monitored.

### 2.4. In Vitro Photothermal Activity of PEG–Starch–IONPs

To evaluate the photothermal activity, the starch–IONP and PEG–starch–IONP suspensions were irradiated with a laser (885 nm, spot size: 5 × 8 mm^2^, MDL-III-885; Changchun New Industries Optoelectronics Tech Co. Ltd., Changchun, China) and the temperature rise was monitored using an IR camera (E5, FLIR Systems, Boston, MA, USA). The assay was performed with (1) varying starch–IONP and PEG–starch–IONP concentrations (0–200 μg Fe/mL) at fixed laser power (1.3 W), and (2) different laser powers (0.8–1.3 W) at a given PEG–starch–IONP concentration (200 μg Fe/mL). In addition, to assess the photothermal stability, the PEG–starch–IONP suspension (100 μgFe/mL) was irradiated with a laser by a switch “on and off” mode at 1 W.

### 2.5. Cellular Analyses of the Photothermal Toxicity by PEG–Starch–IONPs

The U87 MG human glioblastoma cell line was a kind gift from Prof. Sun Young Han (Gyeongsang National University, Republic of Korea). The cell culture was maintained in 10% FBS containing DMEM medium (with 1% antibiotic antimycotic and 1% penicillin–streptomycin) and kept in a cell incubator at 37 °C. To assess the photothermal cytotoxic effects of PEG–starch–IONPs, the U87 MG cells (1 × 10^4^ cells/well) were seeded onto 96-well plates and incubated o.n. The next day, the cells were treated with either PBS or PEG–starch–IONPs (at 100 μg Fe/mL), and then the laser was irradiated to the cells for 10 min at different laser powers (0.8, 0.9. or 1.1 W). After incubation for 48 h, the cells were washed with PBS, and then the relative cell viability was determined by WST-1 assay (iNtRON Biotechnology, Daejeon, Korea).

### 2.6. Animal Studies

All the animal studies were performed according to the protocol approved by the university’s committee for animal research of Gyeongsang National University (GNU-191001-M0047, date of approval: 1 November 2019).

#### 2.6.1. Pharmacokinetics (PK)

The PK profiles of PEG–starch–IONPs were determined using ICR mice (6 weeks old, Hana Co. Ltd., Busan, Korea). After acclimation of the mice in the animal facility for a week, the mice were intravenously (*i.v.*) administered with either starch–IONPs or PEG–starch–IONPs (12 mg Fe/kg) (N = 3). After then, blood samples were collected at pre-determined time points (0, 1, 3, 5, and 30 min post-administration for starch–IONPs, and 0, 0.5, 1, 2, 4, 8, and 12 h for PEG–starch–IONPs). The plasma was instantly separated from the blood by centrifugation (3000 rpm for 3 min) and the Fe contents of the samples were measured by ICP-OES.

#### 2.6.2. In Vivo Photothermal Activity of PEG–Starch–IONPs

U87 MG cells (10^7^ cells per mouse) were implanted in the right-side thigh of athymic nude mice (6 weeks old; Hana Co. Ltd. Busan, Korea). When the average tumor size reached 200 mm^3^, the mice were administered with either starch–IONPs or PEG–starch–IONPs by *i.v.* injection (24 mg Fe/kg). At 4 h post-administration, the mice were anesthetized (with *i.p.* injection of ketamine/xylazine), and then a laser was irradiated to the tumor region for 10 min at varying laser powers (0.6–1.0 W) (N = 3). The temperature of the tumor region was measured with an IR camera (E5, FLIR Systems).

#### 2.6.3. Assessment of the Tumor Accumulation of PEG–Starch–IONPs

When the average tumor size reached 200 mm^3^, the U87 MG *s.c.* xenograft tumor-bearing nude mice were divided into the following 3 groups (N = 5): (1) PBS control, (2) starch–IONPs, and (3) PEG–starch–IONPs and administered with the samples (24 mg Fe/kg) by *i.v.* injection. At 4 h post-administration, the mice were euthanized, and the tumors were harvested. The tumors were then digested by incubation in concentrated hydrochloric acid (HCl) and the Fe contents of the particles were quantified by ICP-OES.

#### 2.6.4. Tumor Growth Inhibition by PEG–Starch–IONPs-Based PTT

When the average tumor size reached 100 mm^3^ (day 1), the U87 MG *s.c.* xenograft tumor-bearing nude mice were divided into the following 4 groups (N = 5): (1) PBS control, (2) starch–IONPs + PTT (starch–IONPs: 24 mg Fe/kg), (3) PEG–starch–IONPs (24 mg Fe/kg), and (4) PEG–starch–IONPs + PTT (PEG–starch–IONPs: 24 mg Fe/kg). For groups 2 and 4, at 4 h post-administration, a laser was irradiated for 10 min at the tumor region (at a power of 0.9 W) after anesthetizing the mice with ketamine/xylazine. The size of the tumors was monitored daily using a vernier caliper. The tumor size was calculated with the formula *V* (mm^3^) = (*a*^2^ × *b*)/2. *V* is tumor volume, *a* is tumor width, and *b* is tumor length. The study was terminated when the average tumor volume of the control mice reached 1000 mm^3^.

### 2.7. Statistical Analyses

Data are presented as average ± standard error of the mean (SEM). A statistically significant difference among the groups was determined using either Student’s *t*-test or 1-way ANOVA (Tukey’s multiple comparison test as the post hoc test) in GraphPad Prism 5.03 (GraphPad Software; LaJolla, CA, USA).

## 3. Results and Discussions

### 3.1. Physical Characterization of PEG–Starch–IONPs

**Figure 1:**
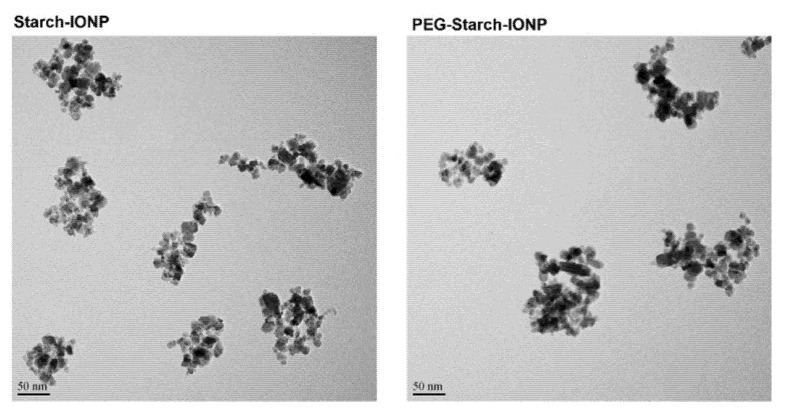
Transmission electron microscopic (TEM) images of starch–IONPs and PEG–starch–IONPs. Scale bar of 50 nm is displayed. (Starch–IONPs: starch-coated iron oxide nanoparticles, and PEG–starch–IONPs: PEGylated starch-coated iron oxide nanoparticles).

**Figure 2:**
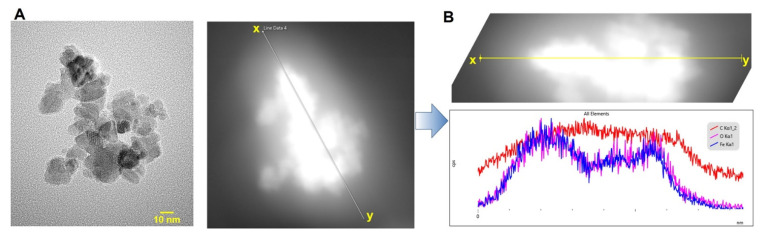
HR-TEM and EDS analysis of PEG–starch–IONPs. (**A**) High-resolution TEM image and (**B**) EDS scan spectrum of the line xy in the (**A**) HR-TEM image.

**Figure 3:**
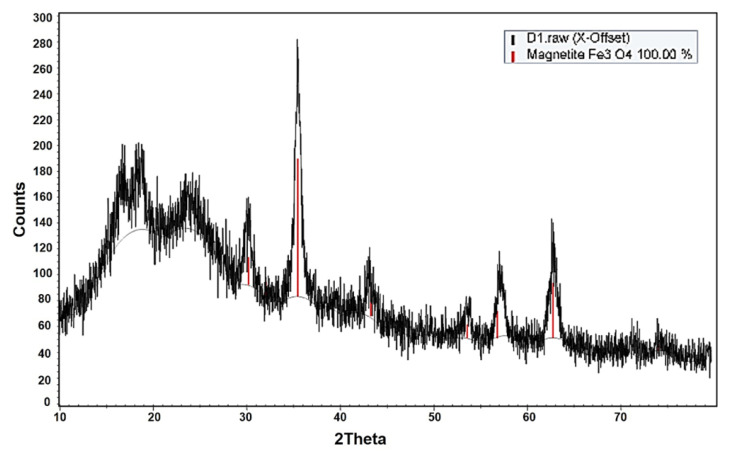
X-ray diffraction (XRD) result of PEG–starch–IONPs. Magnetite peaks are displayed as red in the profile.

Biocompatible polymer-coated IONPs have been of great interest, specifically in the field of theranostic applications [21,36,37,38]. Specifically, starch, a type of natural polymeric sugar, is biodegradable, biocompatible, and non-toxic [39]. Therefore, it has been considered a favored coating material for nanoparticles. Exploiting the hydroxyl groups of the starch coating, starch–IONPs (starch-coated IONPs) could be successfully crosslinked, aminated, and further PEGylated [40]. As shown in Figure 1, the typical core-shell type morphology of starch–IONPs was identified by transmission electron microscopy (TEM), and, importantly, this structure was well-maintained in PEG–starch–IONPs (PEGylated starch-coated IONPs); this implicates that the starch coating could serve as a good protection shield around the hydrophobic IONP core. The structural morphology and elemental map of individual PEG–starch–IONPs were further verified by a high-resolution TEM (HR-TEM) with energy dispersive spectroscopy (EDS) analysis (Figure 2). Based on the HR-TEM images, each spherical nanoparticle of PEG–starch–IONPs were revealed to have the multi-domain core surrounded with shells. The EDS spectrum result of regions of the line xy in the HR-TEM image (Figure 2) demonstrates the existence of magnetite cores of PEG–starch–IONPs. The EDS spectrum shows detectable amounts of compositions with Fe, C and O peaks, suggesting that PEG–starch–IONPs are composed of magnetite cores (Fe and O peaks), while C peaks come from shell coatings of PEG–starch. Furthermore, X-ray diffraction (XRD) analysis confirms that PEG–starch–IONPs possess crystalline Fe_3_O_4_ core structures (Figure 3).

The physical properties of PEG–starch–IONPs were characterized for their hydrodynamic size distribution, surface charge (zeta potential), and the amine and PEG contents (Table 1). Based on the TEM images (Figure 1), we could determine the size distribution of individual particles and morphology. Spherical particles, containing magnetite cores, were seen with a diameter around 10 nm in the images in the HR-TEM (Figure 2). The results of DLS analysis (Table 1) show the observed size distribution for the agglomerated particles in the aqueous environment rather than for the individual IONPs [41]. Compared with the starting particles, starch–IONPs, the hydrodynamic diameter slightly increased from 105.2 (±5.9) to 154.4 (±8.3) nm, and the zeta potential changed from −3.3 (±0.2) to +24.9 (±5.1) mV for PEG–starch–IONPs. Slight increases in the hydrodynamic sizes of PEG–starch–IONPs might be related to increased hydration on the surface of the particles by PEGylation [42]. The hydrophilic residues and high surface mobility of PEG has been reported to provide a hydrated steric barrier to stabilize the surfaces of IONPs and disperse particles in the aqueous environment [43]. The average amine content of PEG–starch–IONPs, quantified by ninhydrin assay, was 255 (±28.8) nmol/mg Fe. The average PEG content of PEG–starch–IONPs, determined by barium iodide assay, was 5.3 (±0.4) nmol/mg Fe.

**Figure 4:**
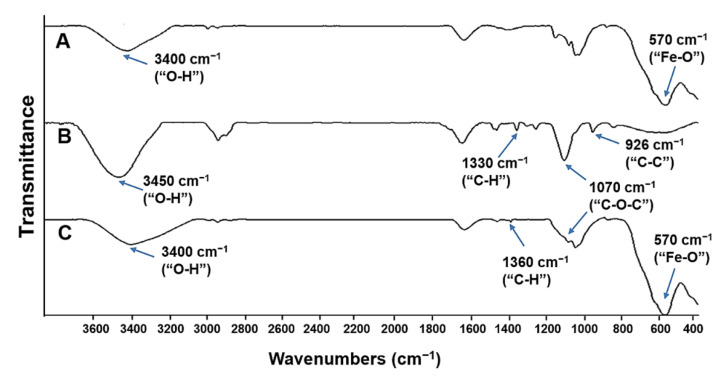
Fourier transform infrared spectroscopy (FT-IR) spectrum of (**A**) starch–IONPs, (**B**) PEG, and (**C**) PEG–starch–IONPs. In each spectrum, the characteristic peaks were displayed with arrows.

**Figure 5:**
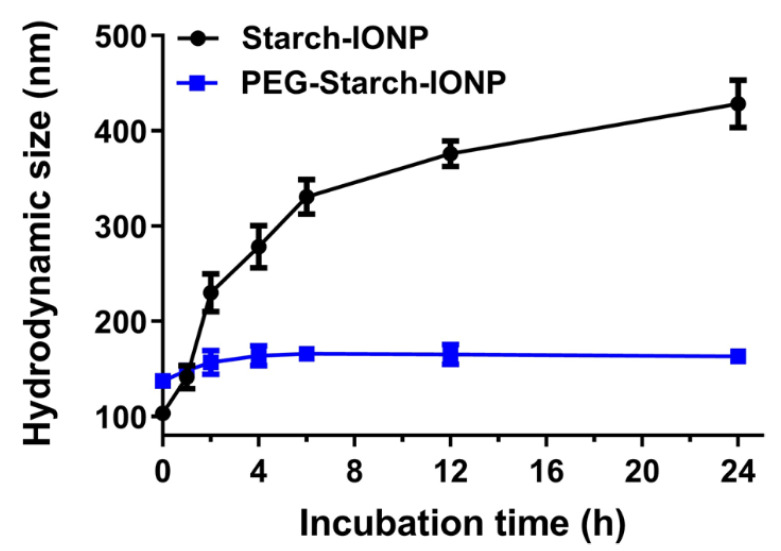
Size stability of nanoparticles. Compared with starch–IONPs, PEG–starch–IONPs could stably maintain their sizes for 24 h.

The successful PEGylation of starch–IONPs was further verified by FT-IR analysis [44]. As shown in Figure 4, in the pure PEG, the characteristic bands were identified, similarly to previous reports [45,46]. (1) the C–C stretching band at around 926 cm^−1^, (2) the C–O–C stretching band at 1070 cm^−1^, and (3) the vibration frequencies at modes of C–C–H, O–C–H, and C–O–H angles at around 1330 cm^−1^. Finally, the hydroxyl groups of PEG provided a broad and intense peak in the range of 3000 to 3500 cm^−1^. In the FT-IR spectrum of PEG–starch–IONPs, vibration frequencies within the iron oxide cores compatible with Fe–O and FeO–H bonds were detected at 542 and 3400 cm^−1^, respectively. The spectrum of PEG did not show the absorption from iron between 500 and 600 cm^−1^. In comparison with starch–IONPs, the representative peak at 1070 cm^−1^ that attributes to the “C-O-C” stretching vibration was observed from both the IR spectra of PEG and PEG–starch–IONPs, suggesting the presence of PEG moieties on the surface of PEG–starch–IONPs. For pure PEG or PEG–starch–IONPs, C–H bending vibrations from the carbon chain were detected in the range of 1200 to 1500 cm^−1^. Lastly, the size stability of PEG–starch–IONPs in plasma was characterized in vitro. As shown in Figure 5, while starch–IONPs showed a gradual and significant increase, PEG–starch–IONPs could stably maintain their size for 24 h. These results were in good accordance with Cole et al. [29].

### 3.2. In Vitro Photothermal Activity of PEG–Starch–IONPs

**Figure 6:**
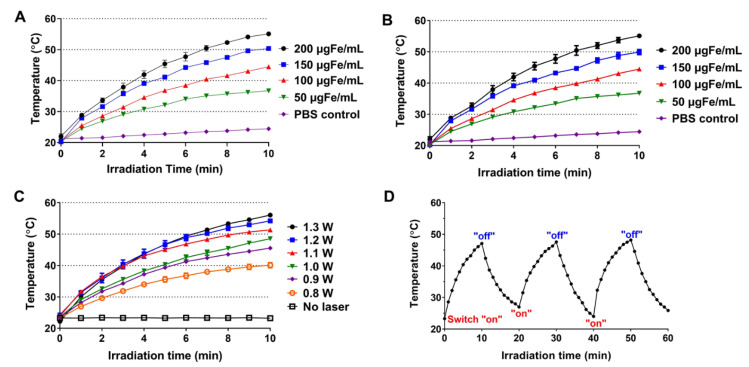
In vitro assessment of the photothermal activity of starch–IONPs and PEG–starch–IONPs. Temperature profiles of particle suspension after laser irradiation with fixed laser power (1.3 W) at varying concentrations (0–200 μg Fe/mL) of (**A**) starch–IONPs or (**B**) PEG–starch–IONPs. Temperature profiles of PEG–starch–IONP suspension irradiated laser (**C**) at a given PEG–starch–IONP concentration (200 μg Fe/mL) with different laser powers (0.8–1.3 W), and (**D**) by a switch “on and off” mode at 1 W with PEG–starch–IONP suspension concentration of 100 μg Fe/mL.

Photo-sensitive metal nanomaterials, such as IONPs, can absorb NIR light and generate heat [7]. By exploiting this photothermal conversion ability, laser-induced selective burning of the tumor (above mid 40 °C) could induce effective cancer cell death [1]. As a PTT agent, the IONPs possess favorable properties such as high photothermal conversion efficiency, ease for surface modification, and feasible magnetic resonance imaging and tumor targeting [7]. To assess the photothermal activity, starch–IONP and PEG–starch-IONP suspensions were irradiated with a NIR laser and the temperature was monitored using an IR camera. The in vitro assay results are shown in Figure 6. As seen with laser irradiation, the suspension temperature gradually increased and slowly reached a plateau (Figure 6A,B). Concentration-dependent temperature rise was observed from both starch–IONP and PEG–starch–IONP suspensions with laser irradiation (Figure 6A,B). The average maximum temperatures after 10 min of laser irradiation were 24.3, 36.7, 44.5, 50.4, 55.1 °C for Starch–IONPs, and 25.8, 37.7, 46.3, 50.1, 56.2 °C for PEG–starch–IONPs, at PBS control, 50, 100, 150, and 200 μg Fe/mL, respectively. These results suggested the PEGylation did not affect the photothermal activity of the nanoparticles. The extent of temperature rise in the PEG–starch–IONP suspension was also positively correlated to the laser power. Increasing the power from 0.8 W to 1.3 W, the maximum temperature of the PEG–starch–IONP suspension increased from 40.1 to 56.1 °C (Figure 6C). Lastly, Figure 6D exhibits the temperature profiles of the PEG–starch–IONP suspension irradiated with a laser by a switch “on-and-off” mode. As seen for every cycle, the PEG–starch–IONP suspension showed a similar pattern of temperature rise and fall, reaching an average maximum temperature of 47.7 °C (at 100 μg Fe/mL concentration of PEG–starch–IONPs and 1 W laser power). These results indicated that, due to photothermal stability, the PEG–starch–IONP-based PTT may elicit reproducible hypothermic effects with repeated laser treatment.

### 3.3. Cellular Analyses of the Photothermal Toxicity by PEG–Starch–IONPs

**Figure 7:**
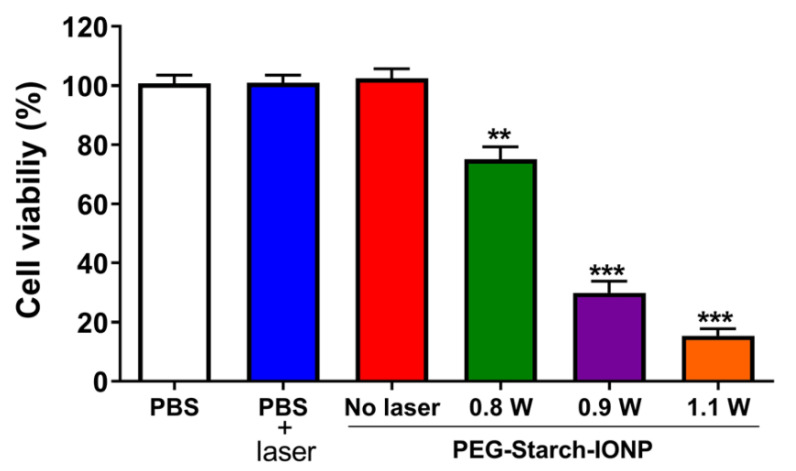
Cytotoxic effects of PEG–starch–IONPs-based photothermal treatment on U87 MG cells. The U87 MG cells were treated with either PBS or PEG–starch–IONPs (at 100 μg Fe/mL), and then a laser was irradiated to the cells for 10 min at different laser powers (0.8, 0.9, or 1.1 W). The relative cell viability was determined by WST-1 assay. The statistically significant differences in the cytotoxicity levels were compared by one-way ANOVA (Tukey’s multiple comparison test as the post hoc test). ** *p* < 0.01, *** *p* < 0.001.

The photothermal cytotoxic effects of PEG–starch–IONPs were assessed on U87 MG cells. The results are shown in Figure 7. As seen, the PEG–starch–IONPs-treated cells showed significantly higher cell death than the laser non-treated groups. The cytotoxicity levels were well-correlated to the laser power. Notably, at temperatures higher than 45 °C (with laser power above 0.9 W), marked cytotoxic effects were observed. The cell viability profiles were 103, 75.1, 29.9, and 15.4% for the PEG–starch–IONP suspensions irradiated with a laser at 0, 0.8, 0.9, and 1.1 W, respectively. In comparison to the PEG–starch–IONPs-treated cells, the PBS-treated cells did not show cytotoxicity even with the laser irradiation at 1.1 W. These results clearly demonstrated the feasibility of PEG–starch–IONPs-based PTT.

### 3.4. Pharmacokinetic Studies

**Figure 8:**
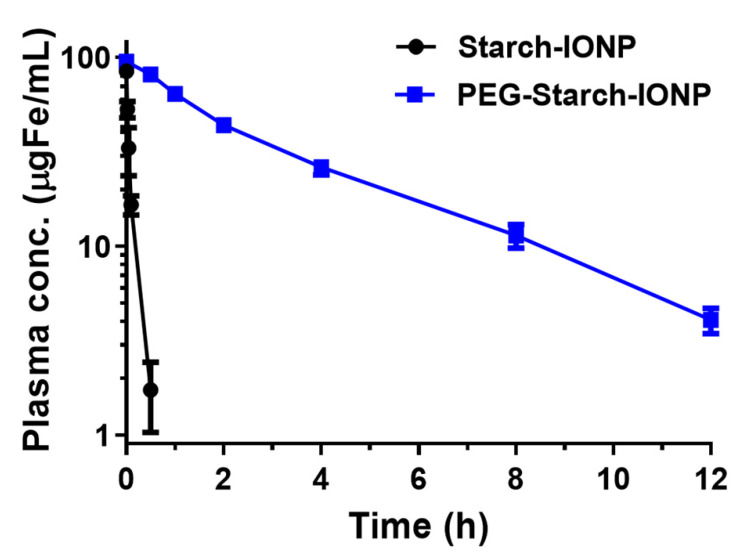
Plasma concentration-versus-time profiles of PEG–starch–IONPs in mice (N = 3). Compared with starch–IONPs, PEG–starch–IONPs exhibited a markedly longer residence in the plasma (plasma half-life: 5.8 min vs. 2.7 h for starch–IONPs and PEG–starch–IONPs, respectively).

The PK profiles of PEG–starch–IONPs were determined in ICR mice. The plasma concentration-versus-time profiles of starch–IONPs and PEG–starch–IONPs are shown in Figure 8. As seen, both starch–IONPs and PEG–starch–IONPs showed a linear decrease in concentration with time; this indicates a good fitting to the one-compartment model. However, there was a great difference in their plasma half-lives. While the plasma half-life of starch–IONPs was 5.8 min, that of the PEG–starch–IONPs was as long as 2.7 h. Notably, these data evidenced that the PEG structure of PEG–starch–IONPs might act as effective shielding against rapid clearance by the reticuloendothelial system (RES), as previously reported by Cole et al. [29]. Compared to dextran- or starch-coated IONPs in previous reports, PEG–starch–IONPs showed extended half-life in plasma in vivo. Feridex^®^ in pharmacokinetic studies using rats showed a blood half-life of 1.35 h [47], and other studies afterwards reported that surface modification of IONPs by polymers enabled the extension of the half-life in plasma [20,29]. PEGylation on starch–IONPs might be effective in preventing plasma opsonization and uptake of particles by macrophages, increasing IONPs circulations in vivo, as evidenced in other reports [48,49].

### 3.5. In Vivo Photothermal Activity of PEG–Starch–IONPs

**Figure 9:**
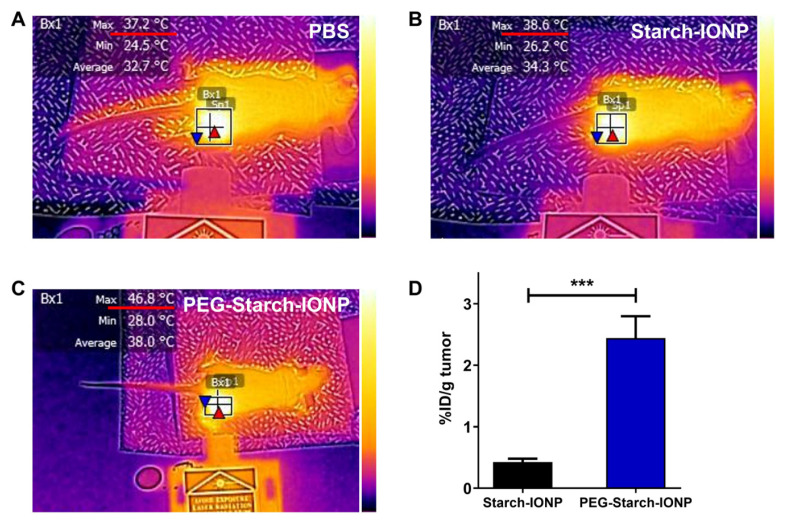
In vivo PTT effects of PEG–starch–IONPs in U87 MG *s.c.* tumor-bearing mice. Representative infrared (IR) images of the mice (N = 3) administered with either (**A**) PBS, (**B**) starch–IONPs, or (**C**) PEG–starch–IONPs during laser irradiation. When the average tumor size reached 200 mm^3^, starch–IONPs or PEG–starch–IONPs were separately administered to mice (N = 3) by intravenous injection (24 mg Fe/mL) and then laser (885 nm) was irradiated for 10 min at varying powers (0.6–1.0 W). The tumor temperature was monitored using an IR camera (E5, FLIR Systems). From the photo images, at 0.9 W the maximum tumor temperatures of the starch–IONPs and PEG–starch–IONPs-administered mice were 38.6 and 46.8 °C, respectively. (**D**) Tumor accumulation profiles of starch–IONPs vs. PEG–starch–IONPs (N = 5). The statistically significant differences in the tumor accumulation profiles were compared by Student’s *t*-test. *** *p* < 0.001.

The photothermal activity of PEG–starch–IONPs was evaluated in U87 MG *s.c.* tumor-bearing nude mice. When the tumor size reached 200 mm^3^, the PEG–starch–IONP suspension (24 mg Fe/kg) was administered by *i.v.* injection and, at 4 h post-administration, a laser was irradiated to the tumor site. The representative IR camera images of the mice irradiated with laser after treatment of PBS, starch–IONPs and PEG–starch–IONPs, are shown in Figure 9A–C, respectively. Also, the maximum tumor temperature profiles are summarized in Table 2. As seen, consistent with the in vitro photothermal activity assay results, the tumor temperature significantly rose by laser irradiation. Furthermore, the maximum tumor temperatures in the IONP-treated mice were positively correlated to the applied laser power (26.6–40.2 °C and 34.2–48.9 °C at 0.6–1.0 W for both starch–IONPs and PEG–starch–IONPs-administered mice, respectively). The tumor temperature achieved by PEG–starch–IONPs was higher than 42.5 °C by clinically available agents, Feridex^®^ or Resovist^®^, coated with dextran [50]. Apparently, the extent of tumor temperature difference was significantly higher in the mice treated with PEG–starch–IONPs than starch–IONPs, suggesting the superiority of PEG–starch–IONPs over starch–IONPs as a PTT agent. As seen from Figure 9D, notably, at 4 h post-administration, the Fe contents in the tumor were 0.43 (±0.1) and 2.44 (0.8) %I.D./gram tumor for starch–IONPs and PEG–starch–IONPs, respectively. Notably, this markedly higher (5.7-fold) tumor accumulation of the PEG–starch–IONPs over starch–IONPs might explain the difference in their photothermal effects. Overall, supported by the results, administration of 24 mg Fe/kg of PEG–starch–IONPs and application of 0.9 W laser power at 4 h post-administration was found to be adequate for further PTT efficacy studies.

### 3.6. Preliminary PEG–Starch–IONPs-Based PTT Efficacy Studies

**Figure 10:**
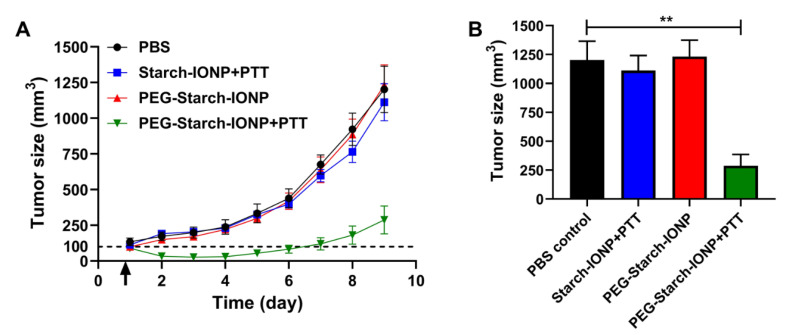
Preliminary efficacy study results (N = 5). (**A**) Tumor growth curves. At day 1 (average tumor size: 100 mm^3^), U87 MG *s.c.* tumor-bearing mice were divided into the following four groups: (1) PBS control, (2) starch–IONPs + PTT (starch–IONPs: 24 mg Fe/kg), (3) PEG–starch–IONPs (24 mgFe/kg), and (4) PEG–starch–IONPs + PTT (PEG–starch–IONPs: 24 mg Fe/kg). For groups two and four, at 4 h post-administration, a laser was irradiated for 10 min at the tumor region (at a power of 0.9 W). The study was continued until the average tumor size of the control group reached above 1000 mm^3^ (day 9). (**B**) Comparison of the tumor sizes at day 9. The statistically significant differences in the tumor sizes were compared by one-way ANOVA (Tukey’s multiple comparison test as the post hoc test). ** *p* < 0.01. (PTT: photothermal treatment).

The feasibility of PEG–starch–IONPs-based PTT was evaluated in U87 MG *s.c*. xenograft tumor-bearing mice. Adopting the optimal experimental conditions (24 mg Fe/kg of PEG–starch–IONPs administration by tail vein injection and laser irradiation at 4 h post-administration at 0.9 W laser power) identified from the in vivo photothermal activity assay, the efficacy studies were carried out with a total of four groups. The tumor growth profiles are displayed in Figure 10A, and the comparison of the tumor sizes at day 9 is shown in Figure 10B. As seen, for the PBS control, starch–IONPs + PTT, and the PEG–starch–IONPs groups, the tumor sizes were exponentially increased at a similar rate. However, in comparison, the PEG–starch–IONPs + PTT group showed significant inhibition of tumor growth and initially even reduction in their tumor sizes. When the study was terminated (on day 9), the average tumor sizes were 1200 (±364), 1110 (±290), 1230 (±315), and 288 (±220) mm^3^ for the PBS control, starch–IONPs + PTT, PEG–starch–IONPs, and PEG–starch–IONPs + PTT, respectively. Notably, the study results demonstrated that a single PTT treatment with PEG–starch–IONPs was sufficient to significantly inhibit tumor growth. Yu et al. reported the therapeutic effects in neuroblastoma by photothermal activity of IONPs modified with mPEG-b-PHEP copolymers, and compared the photothermal therapy by IONP constructs with the combined therapy (photothermal and chemotherapy) [51]. A single PTT treatment by PEG–starch–IONPs was found to be more efficient than IONPs modified with mPEG-b-PHEP copolymers, and compatible to the combined therapy (photothermal and chemotherapy). Overall, through this research, we demonstrated that PEG–starch–IONPs could serve as an effective PTT agent for the treatment of cancer.

## 4. Conclusions

In this research, PEG–starch–IONPs were synthesized and their applicability as a PTT agent was characterized in vitro and in vivo. Notably, through the in vitro assays, PEG–starch–IONPs showed stable and reproducible photothermal effects at repetitive laser irradiation. More importantly, above 0.9 W of laser power, the tumor temperature could be raised to higher than 45 °C necessary for photothermal ablation of the tumor. Compared to dextran- or starch-coated IONPs in previous reports, PEG–starch–IONPs showed an extended half-life in plasma in vivo. Notably, in this study, 5.7-fold higher tumor accumulation was obtained with PEG–starch–IONPs compared to starch–IONPs, finally producing efficient photothermal effects by PEG–starch–IONPs. Based on our results, in vivo tumor ablation could be achieved by administering 24 mg Fe/kg of PEG–starch–IONPs, and applying 0.9 W laser power at the NIR wavelength of 885 nm at 4 h post-administration. Conclusively, the preliminary efficacy study results confirmed that a single PTT with PEG–starch–IONPs was sufficient for effective inhibition of tumor growth.

## Figures and Tables

**Table 1:** Physicochemical properties of starch–IONPs and PEG–starch–IONPs ^1^.

Samples	Hydrodynamic Diameter (nm)	PDI	Zeta Potential (mV)	Amine Content (nmol/mg Fe)	PEG Content (nmol/mg Fe)
**Starch–IONPs**	105.2 (±5.9)	0.11	−3.3 (±0.2)	-	-
**PEG–Starch–IONPs**	154.4 (±8.3)	0.10	+24.9 (±5.1)	255 (±28.8)	5.3 (±0.4)

^1^ Starch–IONPs: starch-coated iron oxide nanoparticles, and PEG–starch–IONPs: PEGylated starch-coated iron oxide nanoparticles.

**Table 2:** Maximum tumor temperature profiles in control, starch–IONPs- and PEG–starch–IONPs-administered U87 MG *s.c*. xenograft tumor-bearing mice after laser irradiation ^1^.

Laser Power (W)	Maximum Tumor Temperature (°C)		
	Control	Starch-IONPs	PEG–Starch–IONPs
**1.0**	38.4 (±3.5)	40.2 (±2.1)	48.9 (±3.3)
**0.9**	36.9 (±3.3)	38.5 (±2.3)	46.6 (±3.4)
**0.8**	33.4 (±2.8)	34.9 (±3.1)	42.3 (±3.1)
**0.7**	28.2 (±1.9)	29.4 (±2.2)	37.8 (±2.5)
**0.6**	25.8 (±1.4)	26.6 (±1.3)	34.2 (±3.4)
**Control**	25.1(±1.2)	25.2 (±1.8)	25.4 (±1.3)

^1^ Starch–IONPs: starch-coated iron oxide nanoparticles, and PEG–starch–IONPs: PEGylated starch-coated iron oxide nanoparticles.

## Data Availability

Not applicable.
